# Mechanisms of *Helicobacter pylori* antibiotic resistance and molecular testing

**DOI:** 10.3389/fmolb.2014.00019

**Published:** 2014-10-24

**Authors:** Toshihiro Nishizawa, Hidekazu Suzuki

**Affiliations:** ^1^Division of Gastroenterology and Hepatology, Department of Internal Medicine, Keio University School of MedicineTokyo, Japan; ^2^Division of Research and Development for Minimally Invasive Treatment, Cancer Center, Keio University School of MedicineTokyo, Japan

**Keywords:** *Helicobacter pylori*, 23S rRNA, gyrA, PBP1, 16S rRNA

## Abstract

Antibiotic resistance in *Helicobacter pylori* (*H.*pylori) is the main factor affecting the efficacy of current treatment methods against infection caused by this organism. The traditional culture methods for testing bacterial susceptibility to antibiotics are expensive and require 10–14 days. Since resistance to clarithromycin, fluoroquinolone, and tetracycline seems to be exclusively caused by specific mutations in a small region of the responsible gene, molecular methods offer an attractive alternative to the above-mentioned techniques. The technique of polymerase chain reaction (PCR) is an accurate and rapid method for the detection of mutations that confer antibiotic resistance. This review highlights the mechanisms of antibiotic resistance in *H. pylori* and the molecular methods for antibiotic susceptibility testing.

## Introduction

*H. pylori* is identified as a Group 1 carcinogen by the World Health Organization International Agency for Research on Cancer (WHO/IARC), and is associated with the development of gastric cancer. Eradication of *H. pylori* infection has been reported as an effective strategy in the treatment of peptic ulcers and gastric mucosa-associated lymphoid tissue lymphoma as well as in the prevention of gastric cancer (Fukase et al., 2008).

Triple treatment including proton pump inhibitor-amoxicillin and clarithromycin or metronidazole proposed at the first Maastricht conference is globally accepted as the technique used to treat *H. pylori* infection. However, recent data show that efficacy of this combination has decreased, with successful cure in only 70% patients (Nishizawa et al., 2014). The 4th edition of the Maastricht consensus recommended a threshold of 15–20% to separate regions of high and low clarithromycin resistance (Malfertheiner et al., 2012). In areas of low clarithromycin resistance, treatments containing clarithromycin are recommended as a first-line empirical treatment. After failure of clarithromycin-containing therapy, either bismuth-containing quadruple therapy or levofloxacin-containing triple therapy is recommended. In areas of high clarithromycin resistance, bismuth-containing quadruple treatments (bismuth subsalicylate, PPI, tetracycline, and metronidazole) are recommended as a first-line empirical treatment. After failure of the quadruple therapy, levofloxacin-containing triple therapy is recommended. After failure of the second line of treatment, subsequent treatment methods should be guided by antimicrobial susceptibility testing whenever possible. The alternative candidates for third-line therapy are rifabutin, fluoroquinolones, tetracycline, furazolidone, and high-dose PPI/amoxicillin therapy (Nishizawa et al., 2008, 2012; Zhang et al., 2014). The traditional culture test for bacterial susceptibility to antibiotics is requires 10–14 days, and is not routinely performed in clinical practice. Minimal inhibitor concentration (MIC)-based individualized treatment for *H. pylori* infection is not prevalent among general practitioners. Molecular techniques for antibiotic susceptibility testing can determine bacterial susceptibility to some antibiotics within a few days. We reviewed the mechanisms of antibiotic resistance in *H. pylori* and the molecular techniques for antibiotic susceptibility testing.

## Clarithromycin

Clarithromycin resistance possibly results from the use of the antibiotic in the pediatric, respiratory, and otorhinolaryngology fields (Kaneko et al., 2004). Global clarithromycin-resistance rates have increased from 9% in 1998 to 17.6% in 2008 in Europe and from 7% in 2000 to 27.7% in 2006 in Japan (Asaka et al., 2010). In patients with clarithromycin-resistant *H. pylori*, it has been reported that the eradication rate achieved with clarithromycin-based regimens shows a marked decrease (Nishizawa et al., 2014). Therefore, the choice of clarithromycin after drug susceptibility testing is promising approach.

The bacteriostatic activity of macrolides such as erythromycin and clarithromycin depends on the capacity to inhibit protein synthesis by binding to the 23S ribosomal subunit (23S rRNA). Extensive studies have demonstrated that point mutations in the peptidyltransferase region encoded in domain V of 23S rRNA are responsible for macrolide resistance. These mutations result in the inhibition of binding between clarithromycin and the ribosomal subunit dedicated to specific antibiotic-related protein synthesis (Versalovic et al., 1996). In particular, the main *23S rRNA* mutations include an adenine-to-guanine transition at positions 2142 (11.7%) and 2143 (69.8%), and an adenine-to-cytosine transversion at position 2142 (2.6%).

Several other point mutations have been identified, such as A2115G, T2117C, G2141A, T2182C, G2224A, C2245T, T2289C, C2611A, and T2717C (Francesco et al., 2011). Besides their low frequency, the clinical relevance of A2115G, T2117C, G2141A, G2224A, T2289C, C2245T mutations is still not proven. The T2182C, C2611A, and T2717C have been associated with low resistance levels (Francesco et al., 2011).

Another relevant mechanism for macrolide resistance is attributed to the efflux pump system. At least 4 conserved families of efflux systems associated with bacterial resistance to antibiotics have been identified (Bina et al., 2000). One of these, widespread among gram-negative bacteria, is the resistance-nodulation-cell division (RND) family of efflux systems (Ma et al., 1995). Bina et al. identified the RND efflux system in *H. pylori. HP0605* from *H. pylori* is a homolog of the *E. coli* gene (*TolC*) encoding the outer membrane protein, TolC, while *HP0607* and *HP0606* and are homologs of the *E. coli* genes *acrA* and *acrB* encoding the membrane fusion and RND cytoplasmic pump protein, respectively (Bina et al., 2000). Kutschke et al. reported that *HP0607* knockout mutants exhibited increased susceptibility to penicillin G, cefotaxime, erythromycin, clarithromycin, tetracycline, clindamycin, novobiocin, and ethidium bromide (Kutschke and de Jonge, 2005). We previously investigated the efficacy of efflux pump inhibitor (Phe-Arg-beta-naphthylamide) in 15 clarithromycin resistant *H. pylori*. In all 15 strains, efflux pump mRNA was expressed, and the MIC of clarithromycin were decreased by using efflux pump inhibitor, despite possessing *23s rRNA* point mutations. In addition, the MIC of clarithromycin was decreased by the efflux pump inhibitor in a concentration-dependent fashion (Hirata et al., 2010).

Most clarithromycin-resistant strains of *H. pylori* have a A2142G or A2143G mutation. Furuta et al. designed the forward primer FP2143G and the reverse primer RP2142G, which specifically anneal to A2143G and A2142G mutated sequences of the *H. pylori 23S rRNA* gene (Furuta et al., 2007). *H. pylori* strains with A2142G, A2143G, and wild-type genotype can be differentiated by amplicon size using the allele-specific, primer-polymerase chain reaction (PCR) method, which is useful and requires only a single PCR run.

Wang et al. reported multiplex sequence analysis (Wang et al., 1999), wherein positions 2142 and 2143 have an AA sequence in wild-type cells, while mutant cells show a change to GA (A2142G) and AG (A2143G), respectively. In the presence of dCTP, dATP, and ddGTP terminating DNA strand elongation results in products of unique length, depending on type of mutation.

Dual priming oligonucleotide (DPO)-PCR is a multiplex PCR assay that increases specificity and sensitivity of detection compared to conventional PCR, blocking non-specific binding sites therefore eliminating imperfect primer annealing. Seeplex^®^ ClaR-*H. pylori* ACE detection (Seegene, Inc., Seoul, Korea) is commercially available DPO-PCR kit to detect *H. pylori* and A2142G, A2143G mutations. Although Seeplex^®^ ClaR-*H. pylori* ACE detection kit does not allow of detection of A2142C mutation, the mutation is less common (<5% of resistant isolates). Lehours et al. reported E-test and DPO-PCR were concordant with regard to clarithromycin susceptibility in 95.3% of the cases (41/43) (Lehours et al., 2011) (Table 1).

**Table 1:** **Commercially available molecular methods for *H. pylori* antibiotics resistance**.

**Product name**	**Seeplex**	**ClariRes**	**GENECUBE**	**HelicoDR**	
Manufacturer (Country)	Seegene (Korea)	Ingenetix (Austria)	TOYOBO (Japan)	Hain Life Science (Germany)	
Assay technique	Dual priming oligonucleotide PCR	Real-time PCR using biprobe	Real-time PCR using quenching probe	DNA strip genotyping test combining PCR and hybridization	
Time-to-result	4 h		30 min	6 h	
Target gene	*23S rRNA*	*23S rRNA*	*23S rRNA*	*23S rRNA*	*gyrA*
Detectable mutation	A2142G, A2143G	A2142G, A2143G	A2142G, A2143G	A2142G, A2143G	87 (Asn to Lys)
		A2142C		A2142C	91 (Asp to Gly, Asn, or Tyr)
*H. pylori* detection	Sensitivity 97.7%	Sensitivity 100%	Sensitivity 100%		
	Specificity 83.1%	Specificity 98–100%	Specificity 100%		
	(Reference test: culture)	(Reference test: culture, RUT, histology)	(Reference test: Hp -IgG, RUT, UBT)		
Comparison with direct sequencing			Sensitivity 100%	Sensitivity 94.9%	Sensitivity 98.2%
			Specificity 100%	Specificity 87.1%	Specificity 80.0%
Comparison with susceptibility test	Concordant rate 95.3%	Sensitivity 82-100%		Sensitivity 94%	Sensitivity 87%
	(41/43)	Specificity 100%		Specificity 99%	Specificity 98.5%

UBT: ^13^C-urea breath test, Hp-IgG: serum anti-H. pylori -IgG, RUT: rapid urease test.

Versalovic et al. developed a method based on rapid restriction analysis of the amplicon obtained from *H. pylori* (Versalovic et al., 1996). Single point mutations at positions 2143, 2142, and 2717 generate specific restriction sites, namely *Bsa*I, *Mbo*II, and *Hha*I, respectively, which can be used in rapid screening for clarithromycin resistance (Masaoka et al., 2004). Fontana et al. developed a new method involving semi-nested PCR and digestion by *Mbo*II, *Bsa*I, and *Hha*I using stool samples. This method is non-invasive and easy to perform (Fontana et al., 2003).

Klesiewicz et al. evaluated the occurrence of A2143G and A2142G mutations in 21 *H. pylori* strains resistant to clarithromycin. The point mutations were detected by PCR followed by restriction fragment length polymorphism (RFLP) analysis. Nine *H. pylori* strains exhibited A2143G mutation and nine *H. pylori* strains exhibited A2142G mutation. The results of RFLP analysis of 3 clarithromycin-resistant strains were negative for both mutations (Klesiewicz et al., 2014).

Wu et al. evaluated the utility of the string test to detect genotypic clarithromycin-resistant *H. pylori* by PCR-RFLP. In the string test, a 90-cm nylon string coiled inside a gelatin capsule was used. A free-end looped string protrudes through a hole in the other end of the capsule. Before the capsule was swallowed, 10–20 cm of the free-end string was pulled out and its position was ensured by adhesion of a small piece of tape to the patient's cheek. It was swallowed with 300 mL of water after 8 h of fasting. One hour after swallowing, the string was retrieved. Approximately 0.5 mL of gastric juice with *H. pylori* attached by every 10 cm of the string was reasonable for molecular biological analysis. Forty three isolates were successfully cultured in 79 patients in whom *23S rRNA* was successfully amplified. Of 5 patients with clarithromycin-resistant *H. pylori*, *23S rRNA* of *H. pylori* isolates from 4 patients could be digested by *Bsa*I. In 38 susceptible isolates, *23S rRNA* of *H. pylori* isolates from 36 patients could not be digested by either *Bsa*I or *Bbs*I. The sensitivity and specificity of the string test to detect genotypic clarithromycin resistance were 66.7 and 97.3%, respectively (Wu et al., 2014).

The restriction enzyme is capable of identifying an A-to-G mutation by the creation of a restriction site, but if an A-to-C mutation occurs, the restriction enzyme may not restrict the DNA at that site. Stone et al. developed a rapid assay based on PCR followed by oligonucleotide ligation for rapid detection of these point mutations, which could differentiate between *H. pylori* strains with A2142G, A2143G, A2142C, and wild–type genotype (Stone et al., 1997).

Several quantitative PCR assays for the determination of clarithromycin susceptibility in *H. pylori* have subsequently been reported. These include a two-step process involving LightCycler PCR for the detection of *H. pylori* followed by melting curve analysis using probe hybridization to detect resistance (Oleastro et al., 2003).

GENECUBE^®^ (TOYOBO Co., LTD. Japan) is a novel, fully automated rapid genetic analyzer capable of extracting nucleic acids from biological material, preparing reaction mixtures, and amplifying the target gene all within 30 min. The amplified target DNA is hybridized with a fluoresce-labeled oligonucleotide (a Qprobe). Upon binding to the target DNA, the Qprobe fluorescence is quenched by the guanine bases in the target. However, the fluorescence reappears as the Qprobe disassociated from the melting target. By detecting this change in fluorescence intensity, A2143G and A2142G mutations are detected. Furuta et al. reported the GENECUBE^®^ genotyping results of the *23S rRNA* gene from gastric tissue samples (*n* = 50) were in complete agreement with those for direct sequencing. Furthermore, gastric juice samples were collected during gastroduodenoscopy in 132 patients. Twenty six of the 132 samples were *H. pylori*-negative based on analysis of serum anti-*H. pylori*-IgG, urease and the ^13^C-urea breath test, and the remaining 106 were *H. pylori*-positive. The GENECUBE^®^ could detect *H. pylori* infection in all patients infected with *H. pylori* based on analysis of serum anti-*H. pylori*-IgG, urease and the ^13^C-urea breath test. Thus, the sensitivity, specificity and validity of the GENECUBE^®^ assay were all 100% (Furuta et al., 2013). GENECUBE^®^ is commercially available, it is not approved for clinical diagnostic use (research use only).

ClariRes^®^ assay (Ingenetix, Vienna, Austria) is a novel commercially available quantitative PCR assay allowing *H. pylori* detection and clarithromycin susceptibility testing in either gastric biopsy or stool specimens. In the biprobe quantitative PCR protocol, followed by hybridization melting point analysis, A2143G, A2142G, and A2142C mutations are detected.

Schabereiter et al. evaluated the clinical usefulness of ClariRes^®^ test in 92 patients who underwent endoscopy. 45 were found to be *H. pylori* infected and invariably were also culture positive. With respect to the detection of *H. pylori* infection, ClariRes^®^ test showed sensitivities of 100% and a specificity of 98%. Of the 45 isolates, 11 were shown to be resistant to clarithromycin by E-test. Compared to E-test, the sensitivity and specificity of ClariRes^®^ test for clarithromycin resistance were 82% and 100% (Schabereiter-Gurtner et al., 2004). Scaletsky et al. evaluated the clinical usefulness of ClariRes^®^ test in Brazilian children. Forty five of the 217 samples were *H. pylori*-positive based on analysis of culture, rapid urease test, or histological examination, and the remaining 172 were *H. pylori*-negative. The sensitivity, specificity and validity of the ClariRes^®^ assay were all 100% for the detection of *H. pylori* infection. In the 45 culture positive patients, the ClariRes^®^ genotyping results of the *23S rRNA* gene from gastric tissue samples were in complete agreement with those for the E-test (Scaletsky et al., 2011).

Can et al. developed a fluorescent *in situ* hybridization (FISH) method to detect *H. pylori* and determine clarithromycin resistance in formalin-fixed, paraffin-embedded, gastric biopsy specimens (Can et al., 2005). Cerqueira et al. evaluated a peptide nucleic acid-FISH method for *H. pylori* clarithromycin resistance detection in paraffin-embedded gastric biopsy specimens. In the retrospective study (*n* = 30 patients), full agreement between peptide nucleic acid-FISH and PCR-sequencing was observed. Compared to the culture followed by E-test, the specificity and sensitivity of peptide nucleic acid-FISH were 90.9 and 84.2%, respectively. In the prospective cohort (*n* = 93 patients), 21 cases were positive by culture. For the patients harboring clarithromycin-resistant *H. pylori*, the method showed sensitivity of 80.0% and specificity of 93.8% (Cerqueira et al., 2013).

Xuan et al. developed an enzymatic colorimetric DNA chip (Xuan et al., 2009) including A2142G, A2142C, A2143G, A2143C, and G2224A mutations, where results were 96.8% (61/63) consistent with those of DNA sequencing. Due to its simplicity and rapidness, the colorimetric DNA chip might be technically feasible for use in general clinical practice.

## Fluoroquinolones

The primary resistance of *H. pylori* to fluoroquinolones has been reported to range between 2–22% in different countries or regions (Suzuki et al., 2010). Resistance to fluoroquinolones is easily acquired, and the resistance rate is relatively high in countries with a high consumption of these drugs (Nishizawa et al., 2006).

Fluoroquinolones exert their antimicrobial activity by inhibiting the function of the enzyme DNA gyrase (Moore et al., 1995). The bacterial gyrase is essential for maintaining the DNA helicoidal structure in addition to being involved in DNA replication, recombination and transcription. The gyrase is a tetramer consisting of two A and two B subunits encoded by the *gyrA* and *gyrB* genes, respectively. The A subunit of DNA gyrase is responsible for DNA cleavage and rejoining, is also the site of action of fluoroquinolones (Matsuzaki et al., 2010). Point mutations in the Quinolones Resistance-Determining Region (QRDR) of *gyrA* prevent binding between the antibiotic and the enzyme, conferring antibiotic bacterial resistance (Nishizawa et al., 2009). *H. pylori* does not possess a gene encoding topoisomerase IV, an important fluoroquinolone target in other bacteria. Point mutations in the QRDR of the *gyrA* gene are mainly at amino acid 87 (Asn to Lys or Tyr) or 91 (Asp to Gly, Asn, or Tyr). We previously reported that the MICs of sitafloxacin in *gyrA* mutation-positive strains differed, depending on the position of the *gyrA* mutation (Suzuki et al., 2009a; Matsuzaki et al., 2012). The MICs were higher in N87-mutated strains (0.21 ± 0.16 μg/ml) than in D91-mutated strains (0.12 ± 0.11 μg/ml, *P* = 0.03) (Matsuzaki et al., 2012). Rimbara et al. proposed that mutation at position 463 in *gyrB* would be a novel mechanism of fluoroquinolone resistance in *H. pylori* (Rimbara et al., 2012).

We previously designed the allele-specific primer, which specifically annealed to the C261A, C261G (87 Asn to Lys), G271A (91 Asp to Asn), G271T (91 Asp to Tyr), and A272G (91 Asp to Gly) mutated sequences of the *gyrA* gene in *H. pylori* (Nishizawa et al., 2007). In the allele-specific PCR method, PCR amplification was performed using allele-specific primers in which the second nucleotide from the 3′ end was designed to match the site of the point mutation.

Glocker et al. developed a reliable fluorescence resonance energy transfer-based quantitative PCR method to detect mutations in the *gyrA* gene (Glocker and Kist, 2004). This method was developed on DNA extracts from *H. pylori* isolates from German patients. Because of the known genetic heterogeneity of *H. pylori* (Suerbaum, 2000), the assay may fail with strains isolated outside Germany, but the test could be altered to adapt to the genetic *gyrA* variants found in different geographical regions.

Cambau et al. developed HelicoDR^®^, a DNA strip genotyping test combining PCR and hybridization (Cambau et al., 2009) that allows the molecular detection of mutations in the *gyrA* gene and 23S rRNA within 6 h. The sensitivity and specificity of detecting resistance using this method were 94 and 99% for clarithromycin and 87% and 98.5% for levofloxacin, respectively. HelicoDR^®^ (Hain Life Science, Germany) is commercially available.

Lee et al. evaluated the clinical usefulness of HelicoDR^®^ test in Korea. Both DNA sequencing after MIC test and HelicoDR^®^ test were performed in *H. pylori* isolates from the gastric mucosa of 101 patients. Among 42 isolates with A2143G mutation by HelicoDR^®^, 83.3% (35/42) of concordance rate was estimated with direct sequencing method and 85.7% (36/42) for MIC test. Among 43 isolates with amino acid 87 (Asn to Lys) mutation by HelicoDR^®^, 71.1% (31/43) of concordance rate was estimated with direct sequencing and 88.4% (38/43) for MIC test. Compared to direct sequencing, the sensitivity and specificity of HelicoDR^®^ test for *23S rRNA* mutation were 94.9 and 87.1%, and those for *gyrA* 98.2 and 80.0%. Compared to MIC test, the sensitivity and specificity of HelicoDR^®^ test for clarithromycin resistance were 55.0 and 80.0% and those for fluoroquinolone were 74.4 and 70.0% (Lee et al., 2004).

## Metronidazole

The prevalence of resistance to metronidazole in *H. pylori* has been reported to range between 8 and 80% in different countries (Suzuki et al., 2010). Metronidazole resistance is much higher in developing countries (more than 60%) than in developed countries (Banatvala et al., 1994). Metronidazole is a prodrug that needs to be activated by reduction of the nitro group attached to the imidazole ring. This reduction step leads to the production of DNA-damaging nitroso- and hydroxylamine-containing compounds. The reduction of metronidazole is mainly mediated by oxygen-insensitive NADPH nitroreductase (RdxA), NADPH-flavin-oxidoreductase (FrxA), and ferredoxin-like enzymes (FrxB) in *H. pylori* (Francesco et al., 2011). Different mutations involving the *rdxA* gene have been identified in metronidazole-resistant strains (Masaoka et al., 2006). In the *rdxA* gene, complex genetic events (insertions and deletions of transposons, and missense and frameshift mutations) could be simultaneously present. These mutations are recognized as the main mechanism conferring metronidazole resistance in *H. pylori.* Point mutations in *frxA* and *frxB* can increase bacterial resistance exclusively in the presence of mutations in the *rdxA* gene. We previously demonstrated a novel mechanism of metronidazole resistance in *H. pylori*, namely, aberrant increase of superoxide dismutase expression resulting from the mutation of the ferric uptake regulator (Fur) (Tsugawa et al., 2011). Superoxide dismutase is essential for protection against superoxide attack. Superoxide dismutase is derepressed by mutant-type Fur, which is associated with the development of metronidazole resistance.

Due to various mutations of *rdxA*, molecular antibiotic susceptibility testing is not applicable for metronidazole.

## Amoxicillin

The prevalence of resistance to amoxicillin has fortunately remained low, where most studies have reported it as less than 2% in all countries, except in Bangladesh (6.6%) (Nahar et al., 2004). Amoxicillin acts by interfering with peptidoglycan synthesis, especially by blocking transporters, namely penicillin binding proteins (PBP). Multiple mutations in *pbp1* gene are the major reason for amoxicillin resistance. Although amoxicillin resistance in *H. pylori* is rare, we previously reported that the MIC_90_ of amoxicillin showed a 2-fold increase with the failure of each eradication treatment (Nishizawa et al., 2011a). Low-level resistant strains (MIC: 0.06–0.25 μg/ml) had 0–2 substitutions, while high-level resistant strains had 1–3 substitutions. Low-level resistance to amoxicillin is linked to a point mutation on *pbp1*, and the accumulation of *PBP1* mutations could result in a gradual increase in amoxicillin resistance. Although production of β-lactamase is rare and almost inactive in *H. pylori*, Tseng et al. reported that high-level amoxicillin resistance is associated with β-lactamase production in *H. pylori* (Tseng et al., 2009). Due to different mutations of *PBP1*, molecular antibiotic susceptibility testing is not applicable for amoxicillin.

## Tetracycline

The prevalence of resistance to tetracycline also has fortunately remained low; it was reported to be less than 2% in most studies (Suzuki et al., 2010). Tetracyclines are bacteriostatic drugs that exert their antimicrobial effect on the 30S subunit of the ribosome and block the binding of aminoacyl-tRNA, resulting in impaired protein biosynthesis. The resistance of *H. pylori* to tetracyclines is reported to be caused by mutations in the *16S rRNA* (Gerrits et al., 2002). Simultaneous triple point-mutations at positions 965–967 are recognized to be major responsible for tetracycline resistance. Levels of resistance are proportional to the number of changes in the AGA 965–967. Single and double point-mutations are associated with low and intermediate MIC values, respectively. High resistance levels are observed in the substitution of an AGA with a TTC triplet.

Ribeiro et al. developed a PCR-RFLP assay allowing rapid and reproducible identification of mutations mediating high-level tetracycline resistance in *H. pylori* (Ribeiro et al., 2004). The substitution of an AGA with a TTC triplet creates an additional *Hinf*I restriction site. This PCR-RFLP assay distinguishes high-level tetracycline resistant isolates from low-level tetracycline resistant and tetracycline susceptible *H. pylori* strains.

Glocker et al. developed real-time PCR to detect *16S rRNA* gene mutations that was capable of differentiating between wild-type strains and resistant strains exhibiting single-, double-, or triple-base-pair mutations (Glocker et al., 2005). Future studies need to address the question of whether additional mutations play a role in the resistance of *H. pylori* to tetracycline.

## Rifabutin

The mean *H. pylori* rifabutin-resistance rate (calculated from 11 studies including 2982 patients) was 1.3% (Gisbert and Calvet, 2012). When only studies including patients naïve to *H. pylori* eradication treatment were considered, this figure was even lower (0.6%). We previously investigated the resistance to rifabutin of *H. pylori* isolated from both general hospital and a hospital specialized for chronic respiratory disease, including pulmonary tuberculosis. Among 94 strains tested, 7 (7.4%) were isolated from patients with a past rifampicin treatment. All these 7 strains showed high rifabutin resistance (Suzuki et al., 2009b). Rifabutin is an antituberculous agent derived from rifamycin-S, which is structurally similar to rifampicin. Rifabutin inhibits the expression of beta-subunit of DNA-dependent RNA polymerase of *H. pylori*, which is encoded by the *rpoB* gene (Nishizawa et al., 2011b). Rifabutin-resistant isolates of *H. pylori* showed mutations in codon 149, codons 525 to 545, or codon 586 (Heep et al., 2002). Due to the different mutations of *rpoB*, the molecular antibiotic susceptibility testing is not applicable for rifabutin.

## Conclusion

Conventional methods used to assess the level of antibiotic-resistance of *H. pylori* are culture-based methods used in combination with agar dilution or the E-test. However, because of the slow growth and particular requirements of *H. pylori* culture, this approach is not reliable for use in most routine clinical laboratory. Since resistance to clarithromycin, fluoroquinolone, and tetracycline seems to be a result of specific mutations in a small region of the responsible gene, molecular methods offer an attractive alternative. Some reliable molecular methods are commercially available. However, large-scale prospective studies should be performed to assess the full clinical potential of these molecular methods and its economic feasibility. Users should keep in mind that whenever possible *H. pylori* culture should be performed and only in cases where standard microbiology fails, the use of molecular methods are really indicated.

### Conflict of interest statement

The authors declare that the research was conducted in the absence of any commercial or financial relationships that could be construed as a potential conflict of interest.
